# Distribution and fate of HCH isomers and DDT metabolites in a tropical environment–case study Cameron Highlands–Malaysia

**DOI:** 10.1186/1752-153X-6-130

**Published:** 2012-11-07

**Authors:** Naghmeh Saadati, Md Pauzi Abdullah, Zuriati Zakaria, Majid Rezayi, Nader Hosseinizare

**Affiliations:** 1 School of Chemical Sciences and Food Technology, Faculty of Science and Technology, Universiti Kebangsaan of Malaysia, Bangi, Malaysia; 2 Water, Soil and Sediment Laboratoy Center, Khuzestan Water and Power Authority, Ahvaz, Iran; 3 Malaysia-Japan International Institute of Technology, Universiti Technologi Malaysia, Jalan Semarak, Kuala Lumpur, Malaysia

**Keywords:** HCHs, DDTs, POPs, Tropical environment, Cameron highlands

## Abstract

**Background:**

The serious impact effects of persistent organic pollutants such as organochlorine pesticides, especially dichlorodiphenyltrichloroethane family (DDTs) and hexachlorocyclohexane isomers (HCHs) have been causing widespread concern, despite effective control on their manufacturing, agricultural and vector practices. In that, in addition to the previous global limitations on DDTs usage, α-HCH, β-HCH and lindane have also became an on-going topic of global relevance based on the latest Stockholm Convention list on 10th of May 2009. Concentrations of dichlorodiphenyltrichloroethane family (DDTs) and hexachlorocyclohexane isomers (HCHs) were determined by GC-ECD in Cameron Highlands, the main vegetables and flowers farming area in Malaysia as an agricultural tropical environment. A total of 112 surface water and sediment samples at eight points were collected along the main rivers in the area namely Telom and Bertam in the dry and wet seasons of 2011.

**Results:**

Total concentration of HCH isomers ranged from not detected to 25.03 ng/L in the water (mean of 5.55 ±6.0 ng/L), while, it ranged from 0.002 to 59.17 ng/g (mean of 8.06±9.39 ng/g) in the sediment. Total concentration of DDT and its metabolites in the water samples varied from not detected to 8.0 ng/L (mean of 0.90±1.66 ng/g), whereas, it was in the range of 0.025 to 23.24 ng/g (mean of 2.55±4.0 ng/g) in the surface sediment samples. The ratio of HCHs and DDTs composition indicated an obvious historical usage and new inputs of these pesticides. Among alpha, beta, gamma and delta isomers of HCH, gamma was the most dominant component in the sediment and water as well. Some seasonal variations in the level of selected pesticides were noted.

**Conclusions:**

The results illustrate distribution, behaviour and fate of HCHs, and DDTs have closely connected with topological and meteorological properties of the area beyond their chemical characterizations. The features of environmental circumstances exceed one or more of these characters in importance than the other. Although the results show that the situation is better than 1998, the impact of persistent agrochemicals such as lindane and 4,4′DDE are revealed in a key tropical area of Malaysia.

## Background

Persistent organochlorine pesticides (OCPs), especially dichlorodiphenyltrichloroethanes (DDTs) and hexachlorocyclohexane isomers (HCHs) cause widespread concern, despite controls on their manufacturing, agricultural and vector practices. Since OCPs were introduced in the 1940s, they have improved crop yields [1], and have drastically reduced recorded cases of malaria [2]. In agriculture, the estimated consumption value of one of the OCPs, namely lindane, has been reported for various countries in the amount of 287.16 (Europe ), 73.20 (Asia), 63.57 (America), 28.54 (Africa) and 1.03 (Oceania) in thousands of tons [3]. As a result, human health and environment have appeared unexpectedly to experience a number of serious changes of adverse effect following the rise in the use of OCPs [4]. These components are a potential risk as they possess toxic properties, resist degradation, bio-accumulates and persists in terrestrial and aquatic ecosystems. The implementation of the Stockholm Convention on Persistent Organic Pollutants (POPs) in 1974 is probably the significant reason for the reduction in the use of POP pesticides world-wide. However, the distribution and use of some of the OCPs have not ceased completely. Based on the study in China [5] of the fate and simulation of HCH transfer, soil and air are the most receptive media, accepting approximately 40% and 60% of HCH isomers respectively. Therefore, current environment suffers greatly from the historical application effects of OCPs, as well as from new inputs [4,6-9]. Various new inputs of HCHs have been reported annually in different regions around the world. Hence, there is evidence on illegal pesticide usage; Chen et al. [10] and the other researchers have reported new inputs of lindane (γ-HCH) in Asia [11-17]. The OCPs founded amounts in rivers and lakes in different part of the world especially nearby the study area, Aisa, has been gathered in Table 1. They researchers clearly pointed out that human activities are the main source of contamination by OCPs, which almost certainly relates to activities such as agricultural chemicals, domestic and industrial discharge, street and road run-off, car exhausts and slum sewage, and other strongly related factors including soil erosion caused by deforestation as well as atmospheric transport [18]. The 4th meeting of the Conference of Parties agreed to put α-HCH, β-HCH and lindane, on the Persistent Organic pollutants (POPs) list at the Stockholm Convention on 26 August 2009 [3].

**Table 1:** Organochlorine pesticide levels in countries nearby the study area and the world

**Area**	**Country**	**Water**	**Sediment**	**Location**	**Reference**
		**ng/L**	**ng/g**		
southeast Asia	India	2.16–567.49	0.92–813.59	Gomti river	[19]
	Thailand		4.12 –214.91	Mae Llong river	[20]
	Thailand	20.87–241.26		Saiburi river	[21]
	Vietnam	<0.5–172.61		red river	[22]
	Vietnam		0.01–110	Gomti river	[23]
the pacific countries	Indonesia	ND–84.94	ND–444.16	Ciliwung river	[24]
	Malaysia	23.4–92.9		Selangor river	[25]
	Singapore	4.90–22.04		coastal marine environment	[26]
east Asia	Japan	0.94–231.8		rivers in Okinawa island	[27]
	china	7.68–615.2	23.11–316.5	Qiantang river	[28]
		16.18–242.4	134–1136	Qiantang river	[29]
		143.3–5104.8	2.43–86.25	Daya bay	[30]
			4.22 – 461	Taihu lake	[31]
		214.4–1819	28.79–52.07	Minjiang river estuary	[32]
	Korea		0.99–14.5	the river estuary	[33]
Other countries	northern Spain		1.8–3.9	coastal environment	[34]
	Nigeria	ND–3726	0.4–43.5	Lagos lagoon complex	[35]

In 2002, Malaysia was a signatory to the Stockholm Convention on POPs and it was committed to carrying out a GEF/UNEP-funded project for the gradual growth of a National Implementation Plan (NIP) for POPs management [36]. In fact, the use of pesticides in Malaysia was not subjected to regulatory control until the Agricultural Chemicals Board was established under the Agricultural Chemicals Act 1974. The use of persistent OCPs was then gradually controlled by a series of governmental rules. This policy led to their controlled use in the mid 1970s.

Historically, pesticides have been used to enhance the crop yields in Malaysia since the Second World War [37]. The country became a model for the World Health Organization (WHO), following the successful control of malaria mosquito vectors by DDT during the 1950s. [17]. Most of the regulated pesticides under the Pesticide Act 1974 were used in the sector until they were banned in the late 1990s [38]. Despite that residues of these pesticides have been frequently found in various media of the environment such as water, sediment and biota [18,36,39].

Md. Sani [38] pointed out that there is no integrated programme to monitor pesticides compared with other hazardous chemicals. Tropical rainforest areas, such as Malaysia are traditionally agriculture-based countries. In peninsular Malaysia, Cameron Highlands is a tourist resort which is the second most important state for growing vegetables (mostly cabbage, tomato, and leafy vegetables), and are also important for tea, flowers and fruit. In addition, Cameron Highlands Catchment area is a source of water supply to many areas of Peninsular Malaysia [40]. Sg.Telom, Sg.Bertam and Sg.Lemoi are the three main rivers of the Cameron Highlands Catchment, which drains the northern, middle and southern sections of the highlands. Over the years, the condition of the rivers, lake and ponds has been degraded in terms of water pollution, river environment and ecosystem [41]. Based on previous studies, the middle of the Cameron Highlands catchment area with its agriculture and urban pollution sources are the most exposed part of the area. Because of the agricultural activities, the land clearing for development and the road construction, the water quality of rivers has declined [42]. The previous study of Lee et al. [43] showed HCH and DDT levels between 38.3 and 78.3 ng/g and between 19.0 and 113.8 ng/g respectively in the sediment of the Cameron highlands Rivers in 1998. From this study, the risk of contamination by intensive agriculture activities was assumed. Likewise, even though lindane was banned in 2003 in Malaysia [44], there is still evidence of lindane existence in the environment [25,45,46].

This study was performed to investigate the existence and associated risks of organochlorine pesticides namely hexachlorocyclohexane isomers (HCH) and dichlorodiphenyltrichloroethane family (DDT) in the aqueous phase, including water and sediment in the Cameron Highlands Catchment Area.

## Result and discussion

### Topological and meteorological effects

Local topological and meteorological properties play a significant role in OCPs transferring behavior. First of all, the high altitude of 1000 meters at the Blue Valley station, resulted in more chemicals traveling from the soil erosion to the rivers. For instance, as Table 1 indicates, about 70% of sediment particles with sizes between 0.25 and 4 mm, indicate medium sand and pebble, based on the Terazaghi [47] particle size classification. This range of particle size is usually considered as evidence of the origin of the particles being a result of erosion in the study area. Secondly, since rainfall pattern changes, OCP compositions will change too. Based on rainfall data in the Cameron Highlands meteorological station records, February, June and July were assigned as a dry season with 50-150 mm monthly average rainfall and April, May, August and November were considered as a wet season with 300-400 mm. Current results indicated that the mean of t-DDT and t-HCH are higher in the wet season rather than the dry season in Cameron Highlands as reported by Zhou et al. [48]. Zhou et al. [48] suggested that OCPs were released from wet deposits or from soil eroding into water with heavy rain, in the Zhejiang province (east China). Table 2 shows a summary of the OCP distribution found, in terms of minimum, maximum and mean of α-HCH, γ-HCH, β-HCH, δ-HCH, 4,4′DDT, 4,4′DDE and 4,4′DDD at eight stations in the Bertam and Telom rivers in 2011. HCHs are more water soluble and volatile than DDTs; hence HCHs were detected in all the samples and at higher levels the DDTs.

**Table 2:** HCHs and DDTs distribution in eight stations in water (ng/L) and surface sediment (ng/g) of Bertam and Telom rivers-2011-Cameron Highlands-Malaysia

		**4,4′-DDE**	**4,4′-DDD**	**4,4′-DDT**	**Alpha HCH**	**Gamma HCH**	**Beta HCH**	**Delta HCH**
Water	Blue valley	ND-3.30(0.56±1.16)	ND-0.57(0.11±0.21)	ND	ND-0.14(0.06±0.06)	1.25-4.21(2.69±1.06)	0.175-0.38(0.27±0.07)	0.01-0.79(0.32±0.34)
	Telom	ND-1.28(0.19±0.45)	ND-0.47(0.07±0.16)	ND-0.32(0.05±0.11)	ND -1.44(0.99±0.61)	1.72-2.33(1.93±0.19)	0.22-0.24(0.23±0.01)	0.24-0.26(0.25±0.01)
	golf field	ND-1.01(0.14±0.36)	ND-1.47(0.21±0.51)	ND-0.94(0.15±0.35)	ND-0.38(0.36±0.01)	0.30-6.52(2.50±2.40)	0.51-1.95(0.96±0.52)	0.62-0.95(0.81±0.01)
	taman sedia	ND-2.38(0.4±0.91)	ND-3.29(0.55±1.25)	ND-2.35(1.17±1.28)	ND-1.00(0.96±0.04)	0.44-7.66(3.43±2.05)	0.56-1.17(0.81±0.24)	0.53-2.09(1.53±0.72)
	fama office	ND-0.56(0.14±0.24)	ND-0.66(0.11±0.25)	ND-0.02(0.02±0.01)	ND-2.78(2.13±0.54)	0.02-18.53(6.52±6.48)	0.715-1.46(1.15±0.26)	0.74-3.67(1.64±1.21)
	parti fall	ND-0.54(0.15±0.23)	ND-0.99(0.33±0.46)	ND-0.34(0.09±0.14)	ND-4.35(4.14±0.11)	0.46-18.76(7.41±7.05)	0.020-0.80(0.39±0.27)	0.138-1.85(0.83±0.68)
	BOH tea	ND-1.78(0.30±0.66)	ND-3.18(0.53±1.21)	ND-1.77(0.3±0.67)	ND-0.75(0.40±0.24)	1.79-9.20(3.76±2.37)	0.101-1.17(0.48±0.44)	0.11-2.32(0.58±0.86)
	Habu	ND-2.39(0.66±0.94)	ND-1.76(0.38±0.65)	ND-0.4(0.14±0.18)	ND-0.62(0.59±0.01)	0.51-10.11(5.58±4.26)	0.83-9.87(3.10±3.54)	0.15-1.20(0.77±0.47)
sediment	Blue valley	0.17-1.86(1.06±0.60)	0.17-13.64(4.14±5.21)	0.21-7.92(2.70±3.03)	0.46-1.84(1.03±0.60)	2.71-5.78(4.08±1.03)	0.186-0.72(0.4±0.19)	0.062-1.92(0.62±0.57)
	Telom	0.07-2.39(1.18±0.77)	0.10-1.84(0.90±0.52)	0.048-1.37(0.82±0.39)	0.0354-1.06(0.49±0.43)	1.80-30.73(11.63±9.38)	0.052-0.97(0.34±0.34)	0.54-1.78(1.01±0.40)
	golf field	0.14-0.65(0.32±0.17)	0.16-7.00(2.70±2.81)	0.093-1.10(0.45±0.32)	ND-0.24(0.12±0.11)	1.53-56.40(14.41±17.84)	0.017-1.03(0.44±0.39)	0.26-1.79(0.94±0.63)
	taman sedia	0.09-0.75(0.3±0.19)	0.092-0.75(0.54±0.26)	0.248-1.26(0.86±0.33)	ND-1.96(0.73±0.68)	0.69-23.60(23.60±7.95)	0.011-0.37(0.16±0.12)	0.37-1.51(0.91±0.58)
	fama office	0.011-0.60(0.17±0.21)	0.012-0.49(0.20±0.17)	0.072-1.75(0.73±0.66)	0.015-0.08(0.06±0.02)	0.42-12.98(7.18±5.43)	0.07-0.56(0.25±0.17)	0.12-4.26(1.23±1.53)
	parti fall	ND-0.87(0.19±0.31)	ND-0.55(0.13±0.19)	0.031-1.09(0.26±0.33)	0.044-0.82(0.43±0.24)	0.91-6.10(3.65±1.41)	0.046-0.48(0.18±0.14)	0.06-0.41(0.2±0.12)
	BOH tea	0.014-0.61(0.17±0.20)	0.010-0.32(0.10±0.10)	ND-0.10(0.03±0.04)	1.211-1.36(1.29±0.08)	ND-8.70(1.96±2.69)	0.034-0.97(0.26±0.36)	0.172-1.88(1±0.91)
	Habu	0.18-.76(0.59±0.17)	0.40-4.18(1.71±1.30)	0.104-0.52(0.28±0.14)	0.0132-0.70(0.37±0.29)	1.27-8.51(5.80±2.43)	0.030-0.44(0.22±0.19)	0.01-1.84(0.57±0.70)

sequence of numbers: min-max(mean ± standard deviation), N=112.

ND: not detected.

N: number of samples.

### HCHs in the water and sediment

**Figure 1:**
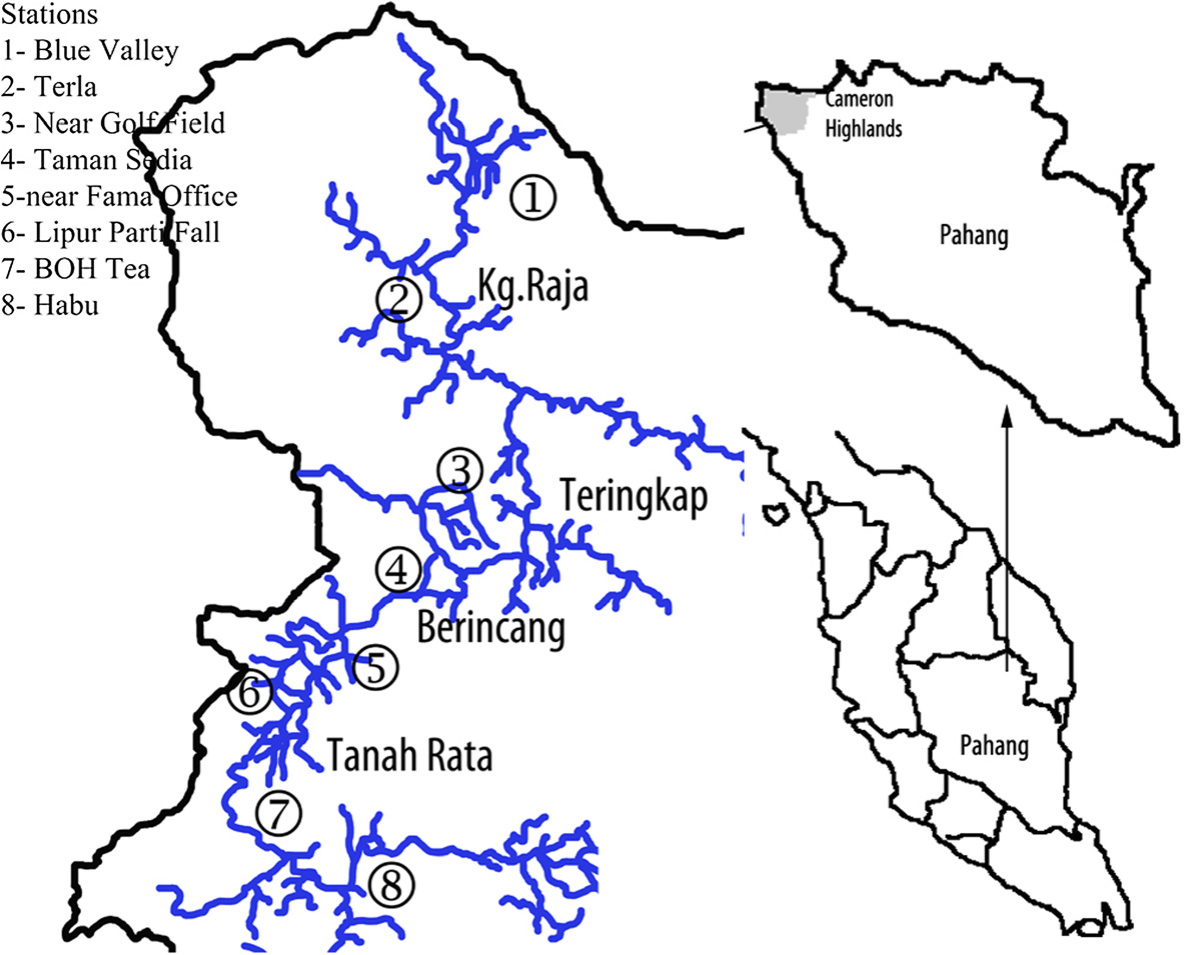
**Map of the Cameron Highlands Catchment and its position in the state of Pahang and Peninsular Malaysia.**The coordination of each sampling station is indicated in Table 1.

Total concentrations of HCHs ranged from not detected to 25.47 ng/L with a mean value of 5.55 ng/L in water (Figure 1), an almost similar level to that detected in the Daliao River, China (3.4-23.8 ng/L) [8]. Higher values of HCHs (ND-113.6 ng/L) in water were reported by Lee et al. [43] These values were lower than in the study of Zhou et al. [29] in the Qiantang River in East China (0.79–202.8ng/L) but higher than those reported for surface water from north-eastern São Paulo, Brazil (0.02–0.6 ng/L) [49].

HCHs in the sediment ranged from not detected to 59.17 (Figure 1), with a mean of 8.06 ng/g, which is quite similar Zhou et al. [30] study (2.43-86.25 ng/g) in Daya Bay China. Higher values of HCHs (19.0-113.8 ng/g) in sediment were reported by Lee et al. [43]. γ-HCH, among HCH isomers was the most abundant in sediment and water. Insecticides containing HCHs are used in agriculture as well as for eradicating mites and lice in human and animals [25].

### DDTs in the water and sediment

**Figure 2:**
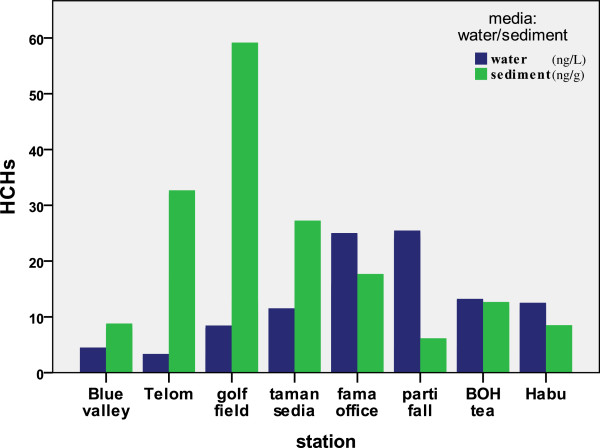
Comparison of total HCH values for eight different stations in the river water (ng/L) and surface sediment (ng/g).

Total concentrations of DDTs ranged from not detected to 7.99 ng/L (Figure 2) in water, which is close to the study of Fernández et al. [50] with 2–6.8 ng/L in the Ebro River (Spain), but it was lower than that revealed by Zhou et al. [48] in the Qiantang River in East China (0.4-97.54 ng/L) but higher than that shown by Rissato et al. [49] in the surface water in the Northeastern part of Sa∼o Paulo State, Brazil (0.02–0.58 ng/L). For the sediments, DDT levels were found in range not detected to 59.19 ng/L (Figure 2). Previous study by Ibrahim [51] reported the levels in the range of 38.3-78.3 ng/g in sediment, in 1998. This means that, although HCHs and DDTs still have the risk of an adverse effect, they have decreased since 1998. DDTs were widely used in the past in Malaysia, and their use was stopped earlier than that of HCHs.

### DDT and HCH composition

**Figure 3:**
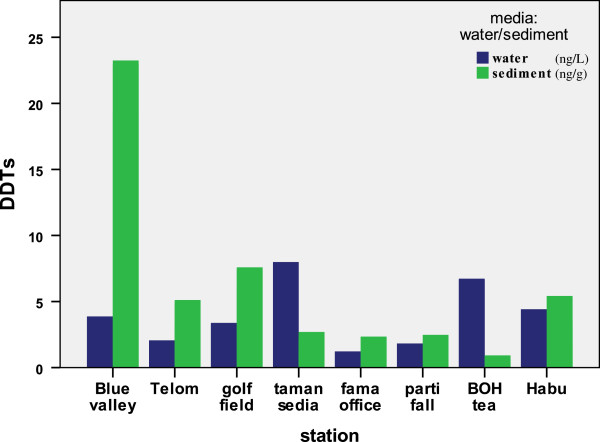
Comparison of total DDT metabolites values for eight different stations in the river water (ng/L) and surface sediment (ng/g).

4,4′DDE, among DDT and its metabolites was the most predominant, with the mean of 0.324 ng/L in water samples (Figure 3). There may be desorption of residual DDT from a contaminated environment, soil desorption from fumigating to control malaria. Since, sediments act as temporary or long-term sinks for OCPs [18] and being hydrophobic OCPs can simply adsorbed to sediments organic matters, thus higher HCH and DDT values were detected. In addition, significant relationship was shown by the HCH and DDT values in the sediment at *P < 0.01* indicating similar source of pollution.

### Seasonal distribution of DDT and HCH

**Figure 4:**
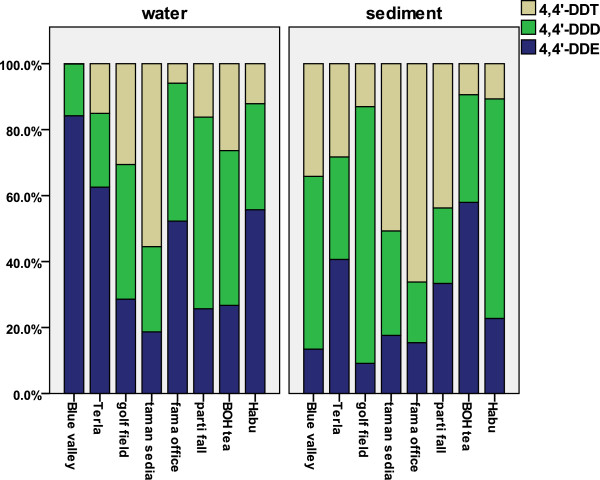
A histogram of DDT metabolites for eight different stations in the river water and surface sediment.

Seasonal distribution shows more DDT and HCH detected during the wet season than in the dry (tables 3 and 4). This could be associated with the surface run-off from the tea and vegetable plantations and with the exposure of the soil to pesticides, or even to more pesticide usage during the wet season, when pesticides wash off more quickly. Mazlan & Mumford [52] pointed out that higher pest infestation in the wet season, resulted in farmer using higher amounts of pesticides. The relationships between rainfall and soil erosion and released OCPs can be found by comparing t-HCH values. The highest amount of t-HCH detected in the water samples was 25.029 ng/L for station number 6 with the most rural and tourist and agricultural activities in August followed by 24.744 ng/L for station No. 5 in July both in the Bertam River (Figure 1). It seems that these components come from vegetable farms and rural pollution sources near to the station and also that these insecticides are able to transfer more in wet weather, as the slope of the area can enhance this transportation. The HCHs are distributed heterogenically. The distributions of HCH and DDT over all period of data sampling are shown in Figures 3 and 4 in the water and sediment samples. Seasonal distributions have some differences which are more significant for water samples. Actually, since rainfall pattern changes, the effect of various contamination sources also changes. A higher rainfall brings mostly contaminations from soil as well as from other point and nonpoint sources. The study area did not experience any month without rainfall. Then the effect of rainfall cannot be neglected completely in the dry season. Another point of course is that wet and dry seasons are not completely separated to each other and in this study the sequence of times studied was dry-wet-wet-dry-dry-wet-wet  .

**Table 3:** HCHs and DDTs seasonal distribution in eight stations in water of Bertam and Telom rivers-2011-Cameron Highlands-Malaysia

		**4,4′-DDE**	**4,4′-DDD**	**4,4′-DDT**	**Alpha HCH**	**Gamma HCH**	**Beta HCH**	**Delta HCH**
dry season	Blue valley	ND-0.64(0.22±0.32)	ND-0.06(0.03±0.04)	ND	0.02-0.02(0.02±0.01)	1.26-4.22(2.89±1.27)	0.25-0.38(0.32±0.07)	0.02-0.53(0.27±0.29)
	Terla	ND-0.05(0.02±0.03)	ND-0.01(0.01±0.01)	ND	ND	1.74-2.07(1.9±0.17)	0.22-0.24(0.23±0.02)	0.24-0.26(0.25±0.01)
	golf field	ND	ND-ND(ND±0)	ND	ND	0.31-1.3(0.66±0.47)	1.94-1.95(1.95±0.01)	0.83-0.89(0.87±0.04)
	taman sedia	ND	ND-ND(ND±0.01)	ND	0.93-1(0.96±0.04)	0.45-7.67(3.56±3.08)	0.56-0.88(0.71±0.15)	2.05-2.1(2.08±0.03)
	fama office	ND	ND-0.01(ND±0.01)	0.01-0.02(0.01±0.01)	1.45-1.51(1.47±0.03)	1.38-18.54(7.69±7.96)	0.72-1.47(1.08±0.37)	0.75-3.67(2.21±1.58)
	parti fall	ND	ND-0.99(0.33±0.49)	ND	ND	0.47-4.38(2.92±1.85)	0.03-0.03(0.03±0.01)	0.14-0.15(0.14±0.01)
	BOH tea	ND-0.02(0.01±0.01)	ND-0.01(0.01±0.01)	ND	0.67-0.75(0.72±0.05)	1.8-9.2(4.69±3.31)	0.85-1.17(0.99±0.14)	0.18-2.32(1.21±1.13)
	Habu	ND-0.02(0.01±0.01)	ND-0.04(0.02±0.02)	ND	ND	0.52-2.7(1.6±1.17)	9.46-9.87(9.72±0.23)	ND
wet season	Blue valley	ND-3.3(0.83±1.49)	ND-0.58(0.15±0.26)	ND-0.01(0.01±0.01)	0.02-0.14(0.08±0.07)	1.92-4.11(2.54±0.91)	0.18-0.27(0.23±0.05)	0.01-0.8(0.39±0.41)
	Terla	ND-1.28(0.33±0.58)	ND-0.47(0.12±0.21)	ND-0.33(0.08±0.15)	0.18-1.45(0.99±0.61)	1.72-2.33(1.96±0.2)	ND	ND
	golf field	ND-1.01(0.26±0.46)	ND-1.47(0.36±0.65)	ND-0.94(0.24±0.42)	0.35-0.38(0.37±0.02)	1.19-6.53(3.89±2.34)	0.51-0.86(0.72±0.13)	0.62-0.95(0.8±0.11)
	taman sedia	ND-2.38(0.6±1.08)	ND-3.29(1.1±1.64)	2.34-2.35(2.35±0.01)	ND	2.39-4.74(3.34±0.87)	0.62-1.17(0.91±0.29)	0.53-1.97(1.26±0.76)
	fama office	ND-0.56(0.19±0.28)	ND-0.66(0.22±0.33)	0.03-0.03(0.03±0.01)	2.18-2.78(2.46±0.27)	0.02-11(5.64±5.34)	1.17-1.3(1.23±0.05)	1.01-1.15(1.09±0.06)
	parti fall	0.01-0.54(0.2±0.25)	0.01-0.97(0.34±0.46)	0.01-0.34(0.13±0.16)	4.04-4.36(4.15±0.11)	3.16-18.77(10.78±7.7)	0.28-0.8(0.49±0.22)	0.36-1.86(1.01±0.65)
	BOH tea	0.02-1.78(0.61±0.86)	0.01-3.19(1.07±1.59)	ND-1.77(0.6±0.87)	0.2-0.28(0.24±0.05)	2.17-4.55(3.07±1.01)	0.11-0.19(0.14±0.04)	0.12-0.21(0.17±0.04)
	Habu	0.02-2.39(1.1±1.01)	0.01-1.76(0.75±0.78)	ND-0.4(0.25±0.19)	0.58-0.62(0.6±0.02)	9.1-10.11(9.58±0.52)	0.83-3.12(1.45±1)	0.15-1.2(0.78±0.47)

sequence of numbers: min-max(mean ± standard deviation) in ng/L. N=56.

ND: not detected.

N: Nomber of sample.

**Table 4:** HCHs and DDTs seasonal distribution in eight stations in surface sediment of Bertam and Telom rivers-2011-Cameron Highlands-Malaysia

		**4,4′-DDE**	**4,4′-DDD**	**4,4′-DDT**	**Alpha HCH**	**Gamma HCH**	**Beta HCH**	**Delta HCH**
dry season	Blue valley	0.18-0.73(0.49±0.25)	0.17-0.82(0.46±0.28)	0.21-1.5(0.9±0.54)	0.51-0.57(0.55±0.1)	2.72-3.65(3.22±0.4)	0.19-0.24(0.22±0.03)	0.07-0.27(0.2±0.1)
	Terla	0.08-2.39(1.49±1.07)	0.11-1.84(1.04±0.76)	0.05-1.38(0.84±0.6)	0.08-1.06(0.67±0.44)	1.81-14.32(6.49±5.62)	0.06-0.97(0.58±0.41)	0.71-1.03(0.86±0.16)
	golf field	0.15-0.65(0.33±0.23)	0.17-0.73(0.37±0.27)	0.1-0.69(0.29±0.28)	0.04-0.2(0.11±0.09)	1.53-10.11(6.52±3.74)	0.08-0.45(0.27±0.2)	0.26-0.45(0.35±0.1)
	taman sedia	0.1-0.75(0.39±0.27)	0.1-0.75(0.52±0.32)	0.25-1.27(0.91±0.49)	0.01-0.46(0.23±0.25)	0.93-3.63(2.44±1.18)	0.02-0.37(0.19±0.19)	0.38-0.42(0.4±0.1)
	fama office	0.19-0.61(0.39±0.22)	0.14-0.49(0.34±0.16)	1.16-1.76(1.46±0.31)	0.09-0.09(0.09±0.1)	0.42-12.9(4.62±6.07)	0.11-0.57(0.33±0.25)	0.12-4.26(2.19±2.27)
	parti fall	0.14-0.87(0.51±0.39)	0.11-0.55(0.32±0.24)	0.04-1.09(0.42±0.48)	0.05-0.82(0.42±0.41)	0.92-6.11(3.42±2.16)	0.05-0.48(0.25±0.2)	0.4-0.41(0.41±0.1)
	BOH tea	0.18-0.61(0.36±0.18)	0.02-0.33(0.15±0.14)	0.01-0.01(0.01±0.1)	1.22-1.37(1.3±0.08)	0.01-8.7(2.89±3.93)	0.04-0.97(0.5±0.51)	1.79-1.88(1.83±0.1)
	Habu	0.19-0.76(0.51±0.25)	0.41-4.19(2.24±1.6)	0.22-0.52(0.38±0.13)	0.02-0.7(0.35±0.37)	1.27-5.38(3.22±2.1)	0.04-0.41(0.21±0.2)	0.2-0.85(0.53±0.35)
wet season	Blue valley	1-1.86(1.5±0.38)	0.97-13.64(6.91±5.47)	0.66-7.92(4.06±3.44)	0.46-1.84(1.41±0.57)	3.2-5.79(4.73±0.87)	0.33-0.72(0.54±0.14)	0.43-1.92(0.95±0.57)
	Terla	0.65-1.49(0.95±0.34)	0.42-1.01(0.81±0.24)	0.59-1(0.82±0.15)	0.04-1.02(0.37±0.41)	3.82-30.74(15.5±9.96)	0.06-0.37(0.16±0.13)	0.54-1.79(1.09±0.47)
	golf field	0.29-0.33(0.31±0.1)	0.47-7(4.46±2.55)	0.21-1.1(0.58±0.31)	0.01-0.25(0.13±0.14)	3.87-56.4(20.34±21.89)	0.02-1.03(0.53±0.45)	0.31-1.79(1.23±0.58)
	taman sedia	0.14-0.32(0.23±0.07)	0.17-0.72(0.56±0.24)	0.59-0.98(0.83±0.15)	0.07-1.97(0.98±0.7)	0.7-23.6(11.89±8.5)	0.11-0.22(0.16±0.1)	1.41-1.51(1.44±0.07)
	fama office	0.02-0.19(0.07±0.08)	0.02-0.3(0.11±0.12)	0.08-0.6(0.26±0.26)	0.02-0.08(0.06±0.1)	2.4-12.99(9.1±4.19)	0.07-0.38(0.22±0.12)	0.23-1.96(0.76±0.73)
	parti fall	0.01-0.1(0.05±0.1)	0.01-0.09(0.05±0.1)	0.1-0.24(0.14±0.06)	0.25-0.6(0.44±0.13)	3.01-4.17(3.82±0.45)	0.09-0.27(0.14±0.08)	0.06-0.22(0.16±0.07)
	BOH tea	0.02-0.06(0.04±0.1)	0.01-0.1(0.07±0.1)	0.01-0.11(0.06±0.06)	ND	0.01-2.23(1.27±0.88)	0.09-0.13(0.11±0.1)	0.18-0.18(0.18±0.1)
	Habu	0.61-0.69(0.65±0.1)	0.43-2.8(1.33±0.93)	0.11-0.34(0.2±0.09)	0.41-0.43(0.42±0.1)	5.12-8.51(7.1±1.28)	0.05-0.44(0.24±0.21)	0.01-1.84(0.6±0.89)

sequence of numbers: min-max(mean±standard deviation) in ng/g. N=56.

ND: not detected.

N: Nomber of Samples.

### HCH isomers frequency and α/γ ratio

The slight differeces in the dry and wet seasons for HCH isomers sequence (table 3 and 4) could be resulted of the effect of rainfall or different pattern of pesticide usage.An increase in α-HCH in the wet season in the water samples especially at the Taman Sedia and Golf course stations, maybe due to the soil erosion at the nearby agricultural areas with previous usage of technical HCH.

γ,β and δ-HCH isomers were detected in most of the water and sediment samples, whereas α-HCH was below the detection level in the majority of the samples (Figure 4). Better transportation of α-HCH than the other isomers and stopping the use of technical may explain this observation. β-HCH, the most persistent isomer of HCH because of its lower vapour pressure, was found to be in higher concentration than other isomers in two of the stations. The level of β-HCH (ranged from 0.916 to 9.713 ng/L) detected at the lower most station, which is the lake of Habu, revealed that most HCHs came from older residues indicating the cumulative effect which appeared in the most persistent isomer of β-HCH at the last station. In contrast, δ-HCH was found most frequently in sediment after γ-HCH.

γ-HCH also known as lindane, has been banned for agriculture use in Malaysia since the last 10 years, was the most predominant isomer found in 75% of the stations. Generally, the most common isomers of HCHs in environment are γ,β and α-HCH, however, γ-HCH and δ-HCH were the most prevalent isomers detected in the most stations in current study. Similar results were reported by Zhou et al. [29] for Qiantang River and Kuranchie-Mensah [53] for Densu River basin.

**Figure 5:**
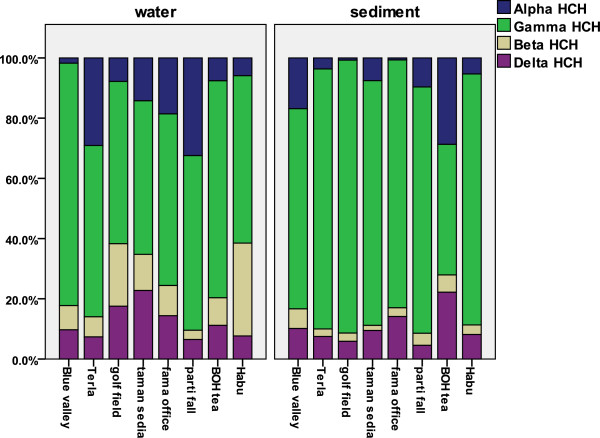
A histogram of composition of HCH isomers for eight different stations in the river water and surface sediment.

Researchers have reported a varying HCH composition with respect to the specific isomers in environmental samples, contrary to the original composition, which leads to facts regarding variations in the behavior of HCHs [54]. Therefore, studies were conducted with regard to individual isomers in order to design the specific ratios that could be used as a tool for differentiation of the origin of pollutants namely as a new one or an old (historical) application. Basically, the α/γ ratio is considered to be helpful in determining the contamination source, based on the difference in ratio from the technical or pesticide origins. Isomeric composition of HCH is generally 60–70% α, 5–12% β, 10–12% γ and 6–10% δ with a 3–7 α/γ ratio [1,8,9]. In this study, α-HCH was below the detection limit in nearly 50% of the samples (water and sediment). For the rest, the α/γ ratio was 0.11 and 0.17 in average terms of sediment and water samples, respectively. The α/γ ratio span is presented in Figure 5 comparing the ratio at various stations in the two media. The result shows that the rivers were faced with new inputs of HCHs because of the higher γ-HCH values compared with the technical HCH composition. It is assumed that, although the usage of HCHs has been increasingly reduced since 1998, there is still HCH usage in the Bertam and Telom Rivers, especially in the Blue valley and BOH tea areas, encompassing tea plantations nearby and golf courses. The Figure 5 suggests the new input and histo rical application pattern of HCHs pesticides. Telom station is more affected by historical applications of HCHs when compared with other stations. Simple data analyses of α/γ ratios over the two seasons show no obvious differences in the sediment samples; meanwhile, they show a clear difference in the water samples (0.07 dry, 0.194 wet). However the ratio of α/γ in water is not stable throughout the seasons, unlike the sediment. This might lead to consideration of the effect of new inputs in the dry season rather than to former usage contamination.

### DDT metabolites frequency and (DDE + DDD)/ DDT ratio

**Figure 6:**
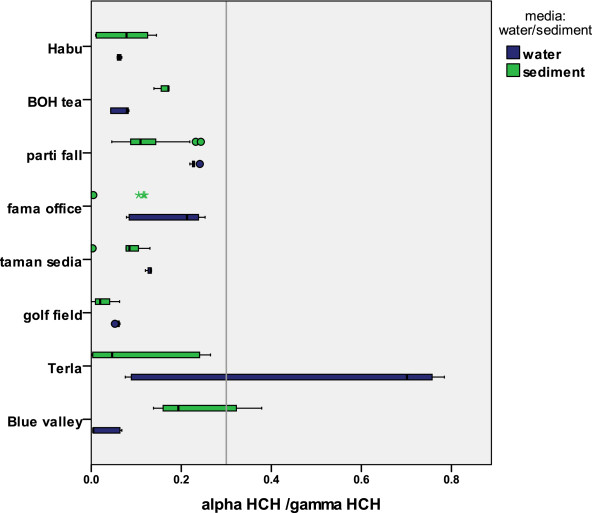
Comparison of variation of α/γ ratio to find new input or historical application sources in the river water and surface sediment between stations.

Most water samples showed the sequence of DDE >DDD >DDT in terms of average for each station during seven times sampling and for sediment samples this sequence was more like DDD> DDT>DDE. It means there is more aerobic metabolite in water and more anaerobic metabolite in sediment. Since ratios of (DDE + DDD)/ DDT > 0.5 indicate long-term weathering, the values in water and sediment are similar. DDT degraded metabolites formed a significant proportion of the DDT compounds. The value of this ratio fell to less than 0.5 in the wet season for some of the sediment samples, evidencing new DDT inputs which is shown in Figure 6.

**Figure 7:**
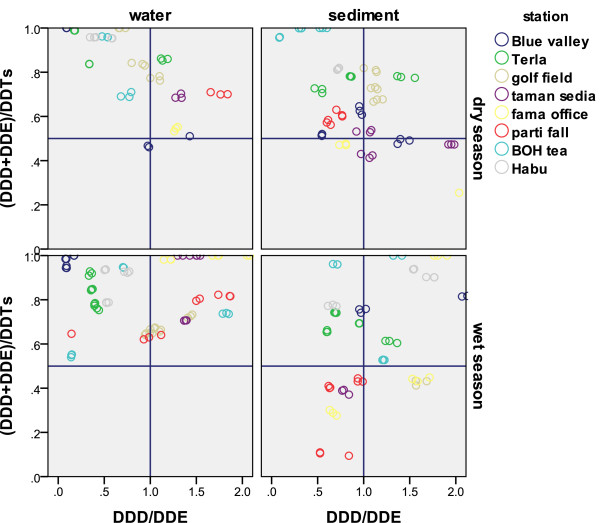
Mean ratio of DDD/DDE and (DDE+DDD)/ΣDDT in the surface water and sediment, wet deposition and dry deposition.

The ratio of DDD/DDE less than 1 indicates aerobic degradation of DDT to DDE, and the ratio values of more than 1 indicate anaerobic degradation of DDT to DDD. Figure 6 presents the ratios of DDD/DDE and DDE + DDD)/DDT simultaneously in one plot. As the figure shows DDE was the most common DDT metabo lite detected in water. This could be evidence of on-going use of DDT because DDT metabolites were either derived aerobically before being transported by surface run-off to the waterway sediment. The other reason might be that DDE has transported better in the atmosphere than other forms [20,29]. The data on different distribution of DDTs in the wet and dry season in water and sediment were showed in the tables 3 and 4. To compare these results with over all period of sampling times, Figure 7 provides the error bar over all samples for water and sediment samples individually based on DDTs metabolites and HCH isomers.

## Conclusion

HCHs and DDTs distribution has closely involved in various main parameters. Beyond the HCHs and DDTs chemical characterizations, the features of environmental circumstances exceed one or more of these parameters in importance. First Malaysia with the features of a tropical and developing country and second Cameron Highland as a forest, massive agricultural, rural, and tourist region and third the times gone by usage of OCPs and finally, the policy of the country to reduce DDTs and HCHs all together make the most pieces of DDTs and HCHs distribution puzzle in the area.

The rainfall pattern caused to produce more HCHs and DDTs from soil erosion from the contaminated area of the past and fairly new usage, mostly in wet season.

γ-HCH and DDE were the most often found components which come from both historical applications and new inputs as well.

The study confirmed degradation of the DDT along the river in the all stations. The degradation to DDE and DDD were 50:50 in dry season meanwhile during the wet season the value of DDE is more, resulting from aerobic degradation of DDT.

HCHs and DDTs residues near and tourist and agricultural activity sites were higher than the other sites far from point source pollutions in the water, even in the sediment samples. The spatial distributions of HCHs in water and sediment were not similar, which reflected in reduced HCHs transportation rate in sediment in comparison with water. The results in this study show no obvious correlation between HCHs residue in the water and sediment, but moderate and even good correlation for gamma HCH at stations closer to pollution sources. Thus, modelling for the HCH residues is complicated due to the historical applications, unknown point sources and the distance from source of pollution in addition to metrological, topological and hydrological specifications of the area. Moreover, it is quite possible to find gamma HCH residue in sheltered cultivation crops, which should be investigated further.

## Methods

### Study area and sampling intervals

**Figure 8:**
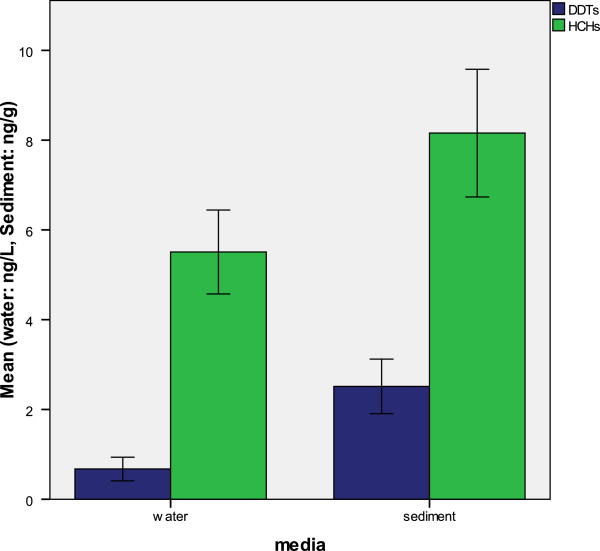
Error Bar diagram of the studied organochlorine pesticides over all water and sediment samples based on the mean of HCHs and DDTs.

The study area, Cameron Highlands, is a district located at 4°28′ N,101°23′, in the north-west of the state of Pahang. Figure 8 shows the study area and the geographical position of sampling points are listed in Table 5. The area is mountainous with 10–35° slopes. It has a moderate temperature of 14-24°C throughout the year with an average rainfall of 2800 mm [42] and no month without rainfall. Most vegetables here are grown at between 900 and 1400 m altitude. Eight sampling sites along the rivers were selected and a total of 112 sediment and water samples were collected from the Bertam and Telom Rivers, upstream to downstream, during two wet and dry seasons. February, June and July were considered as the dry season and April, May, August and November as the wet season in 2011. The sediment samples were collected with a Peterson grab sampler to depth of about 5cm. Water samples were collected straight from the river as the rivers are not that deep, and it could walk across it.

**Table 5:** Stations coordination and mean of physicochemical characteristics of water and particle size of sediment samples

	**Coordination**		**EC**	**Temp**	**TDS**	**Sal**	**Turbidity**	**TSS**	**Particle size sediment**
			**(μs/cm)**	**(°C)**	**(g/l)**	**(%)**	**(NTU)**	**(g/l)**	**mm**
Station 1	N 04 32 998	E 101 24 666	43.3	18.6	5.7	0.02	6	0.291	0.5-2
station 2	N 04 35 329	E 101 25 022	106.3	21.1	23.0	0.05	56	0.443	0.355-1
Station 3	N 04 29 225	E 101 23 065	70.0	19.6	15.4	0.03	68	0.294	0.5-1
Station 4	N 04 28.900	E 101 22.230	68.0	20.0	13.7	0.03	168	0.63	0.5-1
Station 5	N 04 28 580	E 101 22 885	64.3	20.1	12.7	0.03	212	0.673	1-2
Station 6	N 04 28 467	E 101 23 045	61.0	20.6	12.0	0.03	145	0.648	0.7-2
Station 7	N 04 25 871	E 101 23 275	63.3	20.3	11.4	0.03	194	0.366	1-4
Station 8	N 04 26 565	E 101 23 280	67.0	20.6	11.7	0.03	517	0.521	0.25-2

Particle size column present the range of more than 70% of total particles.

### Reagent

The ampoule of mixed of organochlorine pesticide standards consisted of α-HCH, β-HCH, γ-HCH, δ-HCH, 4,4′DDT, 4,4′DDE, 4,4′DDD was obtained from the Supelco (Belle–Fonte, USA). The stock solution (200 ppm) of mixed OCPs was prepared in 10 mL n-hexane. Fresh working standard solutions containing a mixture of the mixed of OCP, surrogates (2, 4, 5, 6-tetrachloro-m-xylene & decachlorobiphenyl) and the internal standard component (pentachloronitrobenzene( was prepared by stepwise dilution of the stock solution with range 1.95, 3.91, 7.81, 15.63, 31.25 and 62.5 μg L^-1^. The sediment samples were collected with a Peterson grab sampler to depth of about 5cm. The sediment samples were wrapped in aluminium foil and stored at 4°C until analysis. 250 g of the sediment was collected from each station to determine particle size. Water samples were collected in glass bottles. The samples were kept at 4°C prior extraction process. A multi-parameter portable device (YSI) was used for onsite measurements of temperature, electrical conductivity, total dissolved solids, salinity and turbidity of the rivers. Organic free water was prepared by passing distilled water through a filter bed containing about 250 g of activated carbon [55,56] and stored in a cleaned narrow-mouth bottle with teflon septa and screw cap. All the glassware was rinsed with an analytical n-hexane before use. All the solvents which were used for extraction, cleanup and enhancement were pesticide grade. The anhydrous sodium sulphate was purified by heating at 400°C for 4 hrs. The florisil (PR Grade) was used for cleanup in an activated form [57].

### Quality control

A gas chromatograph mass spectrometer (GC/MS) analyses were performed on an Agilent 7890A gas chromatograph (GC) directly coupled to the mass spectrometer system (MS) of an Agilent 5975C inert MSD with a triple-axis detector to confirm the order of components. Method Detection Limit (MDL) was found by carrying on a laboratory fortified blank as a real sample. The values for MDL were found between 0.003 and 0.006 μg/L, and Method Quantification Level (MQL) was found between 0.008 and 0.015 μg/L and 0.002 and 0.004 μg/g for water and sediment, respectively. The internal standard concentration was kept constant in all solutions as 100 μg/L. Relative response factor was applied to quantify data. Percentage recoveries were verified by the surrogate component. Surrogate standards were added to each sample to monitor the extraction performance and matrix effects. A recovery value between 75 to 125 percent was considered to quantification and 65 to 135 percent for qualification as well. The concentrations of OCPs were not modified by the recovery ratios of the surrogates. Every sample were analysed in triplicate and the average amount was applied in data analysing.

### Experimental procedures

#### Sediment

The sediment water content was determined by oven drying of about 25 g of wet sediment for 12 h at 105°C. A series of mesh sieves ranged from 0.0125 to 64 mm were applied to determine particle size of the sediment samples. 10.00 g of air dried grounded homogenised sediment sample mixed with 10 g of anhydrous sodium sulphate, which was spiked with 1mL of 0.160 ppm surrogate solutions were extracted with 300 mL n-hexane/acetone 50:50 for six hours in a soxhlet extractor. The extracted volume was reduced by a rotovap to about five mL. This reduced extracted volume was loaded on to the cleanup column filled by 20 g of activated florisil. The cleanup column was eluted three times with 65 mL of n-hexane, 45 mL of 70:30 n-hexane/dichloromethane and 55 mL of dichloromethane. The cleaned up solution was concentrated by evaporating from solvent by use of a rotovap. This solution was further concentrated to 2 mL by a stream of nitrogen. 1 μL litre of the concentrated solution was spiked with 1 μL 100 ppm of internal standard exactly before an injection into the GC-ECD.

#### Water

1000 mL of water sample which it was spiked with 1 mL of 0.080 ppm surrogate solution and added 5 mL methanol was passed through a 6 mL capacity C18 cartridge. The cartridge was optimized with 5 mL ethyleacetate, 5 mL dichloromethane, 10 mL methanol and 10 mL organic free water before use. And it was eluted by 5 mL ethyleacetate and 5 mL dichloromethane. This eluted solution was concentrated by a stream of nitrogen to 1 mL. 1 μL of the concentrated solution was spiked with 1 μL of 100 ppm internal standard exactly before an injection to the GC-ECD.

#### Apparatus

A Varian chromopack CP-3800 Gas Chromatograph was applied to analyse the OCP in the samples. The instrument was equipped with a ^63^Ni electron capture detector and a 30 m × 0.32 mm i.d (0.25 μm film thickness) HP-5ms fused silica capillary column. Nitrogen gas was used as the carrier gas at 1.5 mL/min. The oven temperature was kept at 90°C for 1 minute and increased to 170°C at a rate of 3.5°C/min and then to 280°C at a rate of 5°C/min. The injector and detector temperatures were adjusted at 250 and 300°C respectively. 1 μL of each sample was injected to the GC-ECD for separation and quantitative analysis.

## Competing interests

The authors declare that they have no competing interests.

## Authors’ contributions

These authors contributed equally to this work. All authors read and approved the final manuscript
